# Desires for Individual- and Interpersonal-Level Patient Portal Use for HIV Prevention Among Urban Sexual Minority Men: Cross-sectional Study

**DOI:** 10.2196/43550

**Published:** 2023-02-24

**Authors:** Kevon-Mark P Jackman, Carla Tilchin, Jessica Wagner, Ryan E Flinn, Maria Trent, Carl Latkin, Sebastian Ruhs, Errol L Fields, Matthew M Hamill, Carlos Mahaffey, Adena Greenbaum, Jacky M Jennings

**Affiliations:** 1 Division of Adolescent and Young Adult Medicine Department of Pediatrics Johns Hopkins University School of Medicine Baltimore, MD United States; 2 Center for Child and Community Health Research Department of Pediatrics Johns Hopkins School of Medicine Baltimore, MD United States; 3 Department of Health, Behavior, and Society Johns Hopkins Bloomberg School of Public Health Baltimore, MD United States; 4 Medical College of Georgia Augusta University Augusta, GA United States; 5 Chase Brexton Health Services Baltimore, MD United States; 6 Division of Infectious Diseases Johns Hopkins School of Medicine Baltimore, MD United States; 7 STI/HIV Program Baltimore City Health Department Baltimore, MD United States; 8 Department of Public Health College of Health and Human Sciences Purdue University West Lafayette, IN United States; 9 Department of Epidemiology Johns Hopkins Bloomberg School of Public Health Baltimore, MD United States

**Keywords:** patient portal, HIV, STI, prevention, risk factor, communication, disclosure, digital technology, men who have sex with men, gay, homosexual, sexual minority, African American

## Abstract

**Background:**

Gay, bisexual, and other sexual minority men have expressed the acceptability of patient portals as tools for supporting HIV prevention behaviors, including facilitating disclosure of HIV and other sexually transmitted infection (STI/HIV) laboratory test results to sex partners. However, these studies, in which Black or African American sexual minority men were undersampled, failed to determine the relationship of reported history of discussing HIV results with sex partners and anticipated willingness to disclose web-based STI/HIV test results using a patient portal.

**Objective:**

Among a sample of predominantly Black sexual minority men, this study aimed to (1) determine preferences for patient portal use for HIV prevention and (2) test the associations between reported history of discussing HIV results and anticipated willingness to disclose web-based STI/HIV test results with most recent main and nonmain partners using patient portals.

**Methods:**

Data come from audio-computer self-assisted interview survey data collected during the 3-month visit of a longitudinal cohort study. Univariate analysis assessed patient portal preferences by measuring the valuation rankings of several portal features. Multiple Poisson regression models with robust error variance determined the associations between history of discussing HIV results and willingness to disclose those results using web-based portals by partner type, and to examine criterion validity of the enhancing dyadic communication (EDC) scale to anticipated willingness.

**Results:**

Of the 245 participants, 71% (n=174) were Black and 22% (n=53) were White. Most participants indicated a willingness to share web-based STI/HIV test results with their most recent main partner. Slightly fewer, nonetheless a majority, indicated a willingness to share web-based test results with their most recent nonmain partner. All but 2 patient portal features were valued as high or moderately high priority by >80% of participants. Specifically, tools to help manage HIV (n=183, 75%) and information about pre- and postexposure prophylaxis (both 71%, n=173 and n=175, respectively) were the top-valuated features to include in patient portals for HIV prevention. Discussing HIV test results was significantly associated with increased prevalence of willingness to disclose web-based test results with main (adjusted prevalence ratio [aPR] 1.46, 95% CI 1.21-1.75) and nonmain partners (aPR 1.54, 95% CI 1.23-1.93).

**Conclusions:**

Our findings indicate what features Black sexual minority men envision may be included in the patient portal’s design to optimize HIV prevention, further supporting the criterion validity of the EDC scale. Efforts should be made to support Black sexual minority men’s willingness to disclose STI/HIV testing history and status with partners overall as it is associated significantly with a willingness to disclose testing results digitally via patient portals. Future studies should consider discussion behaviors regarding past HIV test results with partners when tailoring interventions that leverage patient portals in disclosure events.

## Introduction

### Background

Gay, bisexual, and other sexual minority men, and specifically Black or African American (hereafter referred to as Black) sexual minority men, share a disproportionate burden of sexually transmitted infection (STI) incidence, and prevalence, including syphilis, gonorrhea, and HIV [1,2]. Complex intersections of racism, stigma, and sexual behavior are salient drivers of these racial disparities. Sociostructural factors such as incarceration, limitations in access to health care, poverty, educational inequities, under- and unemployment, housing and food instability, and medical mistrust produce conditions that sustain increased STI and HIV (hereinafter, “STI/HIV”) risk factors and incidence of HIV transmission [3-9]. While combating these factors requires a focused policy agenda, Black sexual minority men’s voices must also be incorporated into the design and implementation of innovations to address these disparities [10]. One such innovation is using patient portals to support STI/HIV prevention. Previous research has described preferences for using patient portals to support HIV prevention, including using these portals among sexual minority men to disclose STI/HIV test results to sex partners [11,12]. However, Black participants were undersampled in these studies, leaving a critical knowledge gap concerning their desires and preferences regarding the use of patient portals to support HIV prevention. If left unaddressed, this knowledge gap has the potential to perpetuate the underrepresentation of Black sexual minority men in the development and evaluation of interventions to support their health [10]. Therefore, this study sought to (1) determine preferences for patient portal use for HIV prevention and (2) test the associations between reported history of discussing HIV results with the most recent main and nonmain partner and anticipated willingness to disclose web-based STI/HIV test results to either partner type using patient portals.

### Sexual Health Disclosures Among Black Sexual Minority Men

Advancing knowledge of HIV transmission, continued improvement in antiretroviral therapies (ART), and biomedical approaches to HIV prevention have fundamentally changed the experience of sex, dating, and relationships for many sexual minority men [13]. These advances include pre-exposure prophylaxis (PrEP), postexposure prophylaxis (PEP) for persons at risk for HIV, and ART, which has led to the medical reality that people living with HIV who have achieved sustained viral suppression may finally live free of worry about transmitting HIV if they remain undetectable (also known as U=U or undetectable equals untransmittable) [14]. For sexual minority men at risk for and living with HIV alike, these biomedical advances allow for greater sexual freedom and confidence when engaging sexually with partners of HIV status different from their own [15,16].

Nevertheless, conversations with sexual partners about sexual behavior, sexual health, history of STIs, and ongoing testing habits can remain anxiety-provoking for many sexual minority men. This may be especially true for sexual minority men living with HIV, who may fear rejection, other stigmatizing reactions such as outing and even violence from potential sex partners [16]. Despite the interpersonal risks associated with the disclosure of HIV seropositivity, sexual minority men living with HIV cite their sense of sexual ethics, awareness of laws requiring disclosure of seropositive HIV status before engaging in sex, and other sociohistorical factors that motivate them to share this aspect of their health with sexual partners [16]. Therefore, innovative strategies to support sexual minority men living with HIV in making such disclosures before engaging in sexual behaviors have been identified as an ongoing need [14]. Dynamic web-based apps such as patient portals represent one such innovation with the potential to enhance HIV prevention and care for sexual minority men.

### Dynamic Web-Based Applications

Patient portals are secure websites that give patients convenient, 24-hour access to personal health information from any device connected to the internet [17]. Using a secure username and password, patients can view health information such as laboratory results, immunization history, medications, and recent provider visits [17]. Patients may also be able to request prescription refills, securely message providers, and view educational materials. Patient portals allow patients to receive clear and reliable communication that complements care given in the clinic [18,19]. However, few studies have focused on leveraging the patient portal to deliver patient-facing interventions focused on aspects of HIV prevention and care marked by racial/ethnic inequalities, such as the use of PrEP [20]. The PrEP referral, initiation, use, persistence, and adherence continuum among Black sexual minority men is inhibited due to intersectional stigma, financial cost, health care system inaccessibility, fear of side effects, patient-provider dynamics, competing stressors, and low HIV risk perception [21-23]. In alignment with the National HIV/AIDS strategy, research is needed to develop and implement novel portal-based interventions designed to address the low awareness and uptake of PrEP among populations of Black sexual minority men [24,25].

### Dyadic Communication

Communication of STI/HIV testing information is a critical prevention strategy, but the inability to confirm STI/HIV testing information disclosed by sexual or romantic partners can lower motivations to engage in such preventive communications [26]. The sharing of current information about sexual health can influence decisions by sexual minority men to disclose STI/HIV status, share details about their testing habits, negotiate condom use, or choose other means of sexual pleasure when STI/HIV statuses are unknown or discordant (eg, choosing sexual behaviors that confer a reduced risk of STI/HIV transmission). Patient portals can reduce barriers to engaging in discussions of STI/HIV testing with partners [26]. By exchanging testing information using a patient portal, partners may become aware of what STI/HIV tests were performed, the dates on which they were performed, as well as test results. However, there are little data on interventions that have leveraged patient portals as a pathway to increase discussions around testing and disclosure.

Prior studies suggest that an individual’s score on the enhancing dyadic communication (EDC) scale, a validated scale measuring relative advantages of using patient portals to share STI/HIV test results with sexual partners, has been strongly associated with willingness for patient portal-facilitated test result disclosures [27,28]. Criterion validity refers to the degree to which there is a relationship between a given latent construct score (ie, EDC) and another measure of particular relevance (ie, behavioral intentions) [29-31]. This study examines the criterion validity of the EDC scale and its association with willingness to disclose STI/HIV test results to sex partners using patient portals, among a sample of urban sexual minority men in Baltimore city. In addition, we assess the role of an individual’s history of discussing HIV results with a recent sex partner and its relationship to behavioral intentions to disclose results using web-based patient portals.

## Methods

### Study Overview

The data for this study come from the 3-month visit of a longitudinal cohort study, Understanding Sexual Health in Networks (USHINE), conducted in 2018 in Baltimore, Maryland (MD) [32]. The USHINE Study focused on addressing the local epidemiologic burden of syphilis among Black sexual minority men. The USHINE study participants consist of a convenience sample of sexual minority men aged 18 to 45 years recruited at 2 health department sexual health clinics, a federally qualified health center, a community-based lesbian, gay, bisexual, transgender, and others (LGBTQ+) organization, community engagement events, as well as through respondent driven sampling (RDS). RDS is a peer referral method commonly used when recruiting harder-to-reach populations such as sexual minority men [33]. Individuals were eligible to participate if they reported male sex at birth, current male gender, being aged 18-45 years, having had sex with a man in the past 6 months, residing in Baltimore city, and being willing and able to give informed consent for the study. During baseline and 3-month follow-up study visits, participants completed STI/HIV testing and an audio-computer self-assisted interview (ACASI) survey assessing sexual risk behaviors. This analysis uses data from a convenience-sampled subset of 3-month study visit participants who were presented with ACASI survey questions focused on perceptions about patient portal use related to sexual health.

### Ethical Considerations

Prior to enrollment, study staff met with all participants in a private location to describe the study, review, and obtain written informed consent. Enrolled study participants were compensated US $75 for the baseline visit and US $40 for follow-up visits. The ACASI follow-up survey took an average of 39.5 (SD 17.7) minutes to complete. All study data have been deidentified to protect the privacy and confidentiality of participants. All study activities received ethical review and approval from the institutional review board at Johns Hopkins University (IRB00148799).

### Measures

#### Primary Outcome Variable

To measure participants’ willingness to share web-based STI/HIV test results, the participants were asked (yes or no): if your most recent STI/HIV testing results were available electronically or digitally, would you share the test results with your partner? Questions were asked separately for the participant’s most recent main partner (“a person you have sex with and who you feel committed to above anyone else”) and most recent nonmain partner (“a person you have sex with, even if just one time, but do not feel committed to or don’t know very well”).

#### Primary Independent Variable

Participants were asked to report whether a discussion about HIV test results occurred with their most recent main and most recent nonmain sex partner. Responses included (1) before sex, (2) after sex, (3) before and after sex, or (4) did not discuss. For our analysis, the variable was dichotomized as (1) not discussed and (2) discussed (all other).

#### EDC Scale

The EDC scale consists of 3 survey items measuring participant agreement with beliefs that sharing test results will (1) improve communication on HIV and other STIs, (2) improve confidence in the testing information shared, and (3) help make healthier sexual decisions. EDC survey items were asked separately for main partners and nonmain partners. Item scores range from 0 (strongly disagree) to 3 (strongly agree). Cronbach α was calculated to assess the internal reliability of the EDC scale respective to partner type. A factor score was determined by summation of scores ranging from 0 to 9, and the EDC score was standardized for inclusion in Poisson regression models with robust error variance for willingness to disclose web-based STI/HIV results with partners to obtain unadjusted and adjusted prevalence ratios.

#### Patient Portal Features

Participants were asked about the importance of including features specific to sexual health within a web-based health record (available through a patient portal) as a high priority, moderate priority, low priority, or inappropriate. Features included (1) educational information explaining HIV and other STIs; (2) tips to help talk with sex partners about sexual health; (3) information on ordering HIV and STI home-test kits; (4) information on other locations to get tested; (5) sexual diaries (to help document your sexual experiences); (6) educational games on sexual health; (7) the ability to notify partners of positive results anonymously; (8) the ability to video chat with health care providers; (9) information on social support service linkages (eg, substance use, mental health, and intimate partner violence resources); (10) information about HIV PrEP; (11) information about HIV PEP; (12) information on tools to help manage HIV treatment; and (13) information on the HIV genetic subtype. A free-text “Other” response was included to allow participants to list additional desired features. The comprehensive list of patient portal features was developed during a formative research phase in collaboration with the USHINE Study Community Advisory Board. Members of the Community Advisory Board were Black sexual minority men living in Baltimore.

### Analysis

#### Univariate and Bivariate Analyses

Univariate analysis was conducted to describe the study population and report how participants ranked the importance of patient portal features. Chi-square tests and *t* tests were performed to determine significant within-group differences between participants willing versus those unwilling to share web-based STI/HIV test results with main and nonmain partners by age, race, STI/HIV diagnosis, discussion of HIV test results with recent partners, and EDC score. In addition, the sample mean, SD, and Cronbach α were calculated for prevalent EDC scores respective to main and nonmain partners.

#### Poisson Regression With Robust Error Variance

Unadjusted and adjusted Poisson regression models with robust error variance were used to test the associations between discussing HIV results and willingness to disclose web-based STI/HIV test results with a most recent main and nonmain partner. Poisson regression models with robust error variance have been used in cross-sectional studies with binary outcomes [34]. For model construction, variables were included in the adjusted model if they demonstrated bivariate statistical associations with the outcome variable of a *P* value of <.10. Analyses were conducted using Stata (version 15; StataCorp LLC), and significance was defined as *P*<.05.

## Results

### Sample Characteristics

A total of 567 individuals were recruited and screened for eligibility, of whom 432 (76%) were eligible and 427 (99%) agreed to participate and signed an informed consent form; of them, 353 individuals (83%) completed the 3-month follow-up ACASI survey. Patient portal items were presented to a convenience sample of 253 participants completing the 3-month follow-up study visit ACASI survey and observations with missing data were removed resulting in a final analytical sample of 245 men. Participant ages ranged from 19 to 45 years, with a median age of 29 (IQR 25-34) years. Approximately 72% (n=174) identified racially as Black, and 22% (n=53) identified as White (Table 1). Approximately 20% (n=50) of men reported living with a partner. All participants named both a most recent main partner and a most recent nonmain partner.

Participants living with HIV comprised 37% (91/245) individuals in the study sample; 89% (81/91) participants living with HIV were Black and 5% (5/91) were White. Approximately, 18% (45/245) of the sample received a positive STI diagnosis (syphilis, chlamydia, or gonorrhea) at the 3-month follow-up visit; 38 (84%) of these participants were Black and 4 (9%) were White. Two-thirds (n=163, 67%) of participants reported discussing HIV test results with their most recent main partner, and 136 (56%) reported discussing HIV test results with their most recent nonmain partner (Table 1).

Over three-quarters of the sample (n=190) indicated that they were willing to share web-based STI/HIV test results with their most recent main partner, while 62% (151/245) participants indicated being willing to share web-based test results with their most recent nonmain partner (Table 1). The EDC scale assessed for main partners had a Cronbach α of .94 with a mean sample score of 6.71 (SD 2.59). The EDC scale assessed for nonmain partners had a Cronbach α of .95 and a mean sample score of 5.87 (SD 2.60).

**Table 1:** Descriptive statistics, by willingness to disclose test results for HIV and other sexually transmitted infections with main and nonmain sex partners using patient portals, among sexual minority men in the Understanding Sexual Health in Networks Study, Baltimore, MD, 2018 (N=245).

Variable			Values		Main partners								Nonmain partners							
					Willing		Unwilling		*P* value			Willing				Unwilling			*P* value	
**Total, n (%)**			245		190 (77.5)		55 (22.5)					151 (61.6)				94 (38.4)				
**Age (by quartile group; years)^a,b^, n (%)**										.01										.67
	18 to 25	66 (26.9)		59 (89.4)		7 (10.6)					43 (65.2)				23 (34.8)					
	26 to 29	62 (25.3)		50 (80.6)		12 (19.4)					36 (58.1)				26 (41.9)					
	30 to 34	56 (22.9)		37 (66.1)		19 (33.9)					32 (57.1)				24 42.9)					
	35 to 45	61 (24.9)		44 (72.1)		17 (27.9)					40 (65.6)				21 (34.4)					
**Race/ethnicity^a,b^, n (%)**										.09										.03
	Black or African American	174 (71.0)		129 (74.1)		45 (25.9)					99 (56.9)				75 (43.1)					
	White	53 (21.6)		47 (88.7)		6 (11.3)					41 (77.4)				12 (22.6)					
	Other	18 (7.4)		14 (77.8)		4 (22.2)					11 (61.1)				7 (38.9)					
**Employment^a,b^, n (%)**										.18										.53
	Full-time	126 (52.4)		99 (78.6)		27 (21.4)					83 (65.9)				43 (34.1)					
	Part-time	31 (12.7)		28 (90.3)		3 (9.7)					18 (58.1)				13 (41.9)					
	Unemployed	51 (20.8)		37 (72.6)		14 (27.4)					30 (58.8)				21 (41.2)					
	Other	37 (15.1)		26 (70.3)		11 (29.7)					20 (54.1)				17 (46.0)					
**Living with partner^a,b^, n (%)**										.002										.17
	Yes	50 (20.4)		47 (94.0)		3 (6.0)					35 (70.0)				15 (30.0)					
	No	195 (79.6)		143 (73.3)		52 (26.7)					116 (59.5)				79 (40.5)					
**Living with HIV^a,b^, n (%)**										<.001										<.001
	Yes	91 (37.1)		56 (61.5)		35 (38.5)					40 (44.0)				51 (56.0)					
	No	154 (62.9)		134 (87.0)		20 (13.0)					111 (72.1)				43 (27.9)					
**STI^c^ diagnosis at the 3-month visit, n (%)**										.12				N/A^d^			N/A			.05
	Yes	45 (18.4)		31 (68.9)		14 (31.1)					22 (48.9)				23 (51.1)					
	No	200 (81.6)		159 (79.5)		41 (20.5)					129 (64.5)				71 (35.5)					
**Discussed HIV with main partner^a,b^, n (%)**										<.001										.001
	Yes	163 (66.5)		144 (88.3)		19 (11.7)					112 (68.7)				51 (31.3)					
	No	82 (33.5)		46 (56.1)		36 (43.9)					39 (47.6)				43 (52.4)					
**Discussed HIV with nonmain partner^a,b^, n (%)**										.001										<.001
	Yes	136 (55.5)		116 (85.3)		20 (14.7)					104 (76.5)				32 (23.5)					
	No	109 (44.5)		74 (67.9)		35 (32.1)					47 (43.1)				62 (56.9)					
Main partner EDC^e,f^ score, mean (SD)^g^			6.7 (2.6)		7.1 (2.4)		5.4 (2.6)		<.001			N/A				N/A			N/A	
Nonmain partner EDC score, Mean (SD)^g,h^			5.9 (2.6)		N/A		N/A		N/A			6.5 (2.3)				4.8 (2.6)			<.001	

^a^Chi square used to test group differences.

^b^Row percentages reported for willing versus unwilling comparisons.

^c^STI: sexually transmitted infection.

^d^N/A: not available.

^e^EDC: enhancing dyadic communication.

^f^Cronbach α=.94.

^g^*t* test used to compare group differences.

^h^Cronbach α=.95.

### Patient Portal Features

**Figure 1:**
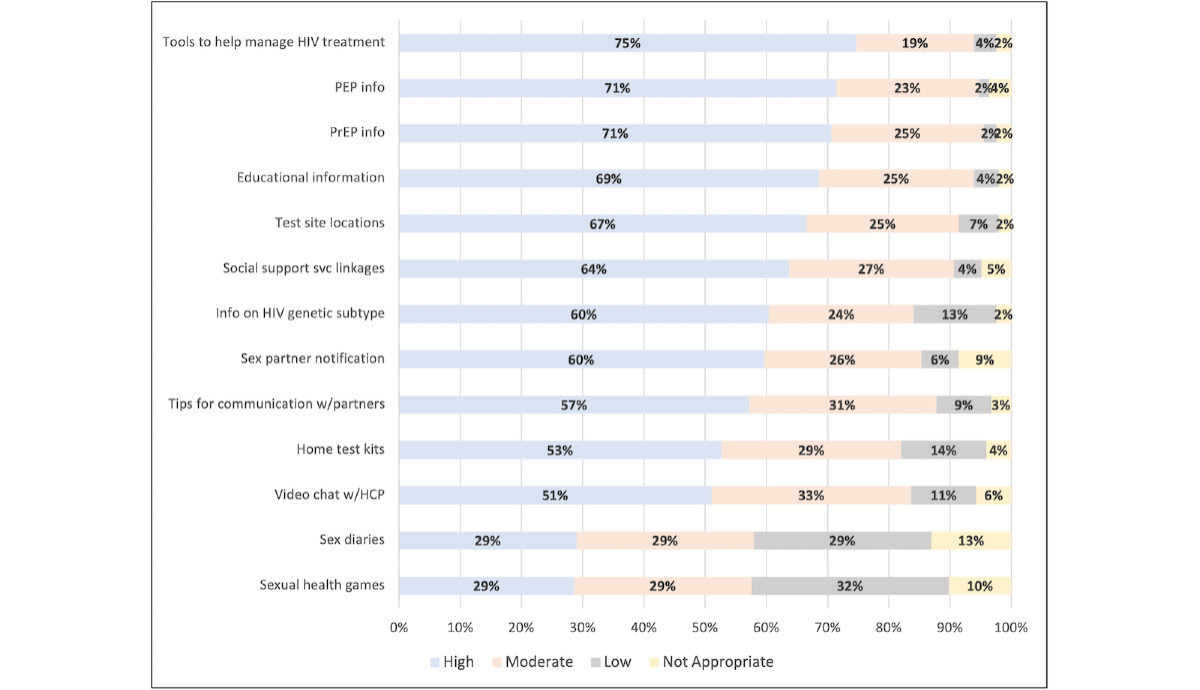
Valuation rankings for patient portal services among sexual minority men in the Understanding Sexual Health in Networks study, Baltimore, MD, 2018 (N=245). HCP: health care provider; PEP: postexposure prophylaxis; PrEP: preexposure prophylaxis; svc: service; w/: with.

All patient portal features were ranked as either high or moderate priority in the range of 82% (n=201) to 96% (n=234) of the population, except for sex diaries to help document sexual experiences and games focused on sexual health (Figure 1). Patient portal features ranked as high priority by the greatest proportion of participants were information on tools to help manage HIV treatment (183/245, 75%), information about PEP (175/245, 71%), and information about PrEP (173/245, 71%).

Anonymous sex partner notification and linkages to social support services were ranked as high or moderate priority among 85% (209/245) and 91% (222/245) participants, respectively. Approximately 206 (84%) participants ranked HIV genetic subtype information in the patient portal as high or moderate priority (Figure 1).

### Bivariate Analysis

#### Differences by Main Exposure Variables

Among participants who reported discussing HIV test results with their most recent main partner (n=163), 144 (88%) were willing to share web-based test results with main partners (Table 1). Among participants who reported discussing HIV test results with their most recent nonmain partner (n=136), 104 (76%) were willing to share web-based results with nonmain partners. The proportion of participants willing to share web-based test results with the partners was significantly greater among those who reported discussing HIV test results with their most recent partners (*P*<.01). Mean EDC scores for main partner were significantly different between participants who were willing versus unwilling to share web-based STI/HIV test results with their main partner (mean 7.1, SD 2.4 vs 5.4, SD 2.6; *P*<.001). Similarly, mean EDC scores for nonmain partner differed between participants willing versus those unwilling to share web-based STI/HIV test results with their nonmain partner (mean 6.5, SD 2.3 vs 4.8 SD 2.6; *P*<.001; Table 1).

#### Differences by Sociodemographic Characteristics

There were significant differences in the proportions of men willing to disclose web-based STI/HIV test records to main partners by age category (*P*=.01; Table 1). Men aged 18 to 25 years were most likely to be willing to disclose (59/66, 89%), followed by men aged 26 to 29 years (50/62, 81%). There were no significant differences by age category in willingness to disclose STI/HIV records to nonmain partners. When looking at race and ethnicity, the greatest difference in the proportion of participants willing to share web-based results was between Black and White participants. Specifically, 74% (129/174) of Black participants versus 89% (47/53) of White participants were willing to share with their main partner (*P*=.09). Similarly, 57% (99/174) of Black participants versus 77% (41/53) of White participants were among those men willing to share with their nonmain partner (*P*=.03). Participants who reported living with a partner were more willing to share web-based STI/HIV results with main partners compared to participants not living with a partner (*P*=.002); however, differences were not significant for willingness to share web-based test results with nonmain partners (*P*=.17; Table 1).

#### Differences by STI/HIV Diagnosis

The proportion of those reporting unwillingness to share web-based test results with their most recent sex partners was significantly higher for those living with HIV (Table 1). Similarly, smaller proportions of participants diagnosed (vs not diagnosed) with a bacterial STI at the 3-month follow-up visit were willing to share web-based test results with both main and nonmain partners. However, differences in willingness to share web-based results by STI diagnosis were only significant for sharing results with nonmain partners (22/45, 49% vs 129/200, 65%, *P*=.05; Table 1).

#### Multivariable Associations With Willingness to Disclose Web-Based Test Results With Partners

Reporting having a discussion about HIV test results with their most recent partner was associated with an increased prevalence of willingness to disclose web-based test results to that main partner (adjusted prevalence ratio [aPR] 1.46, 95% CI 1.21-1.75) and nonmain partner (aPR 1.54, 95% CI 1.23-1.93; Table 1). Similarly, a 1 SD increase in standardized EDC score was associated with a 1.15 (95% CI 1.06-1.25) and 1.27 (95% CI 1.13-1.43) increase in the prevalence of willingness to share web-based test results with their most recent main and nonmain partners, respectively.

Participants aged 18 to 25 years (aPR 0.78, 95% CI 0.62-0.99) and 26 to 29 years (aPR 0.76, 95% CI 0.59-0.99) had a decreased prevalence of willingness to share web-based STI/HIV results with nonmain partners. There was an increased prevalence of willingness to share web-based test results with both a main (prevalence ratio 1.20, 95% CI 1.04-1.36) and a nonmain partner (prevalence ratio 1.36, 95% CI 1.12-1.65) among White participants compared to those among participants of other race and ethnicity categories; however, these associations were not statistically significant in the adjusted model (Table 2). Reporting living with a partner was associated with a 1.26 (95% CI 1.13-1.41) increased prevalence of willingness to disclose web-based STI/HIV with their main partner.

Decreased prevalence of willingness to share web-based test results with their main partner (aPR 0.78, 95% CI 0.67-0.91) and nonmain partner (aPR 0.69, 95% CI 0.55-0.88) was observed among participants living with HIV.

**Table 2:** Unadjusted and adjusted prevalence ratios (95% CI) of willingness to disclose test results for HIV or other sexually transmitted infections with main and nonmain sex partners among sexual minority men in the Understanding Sexual Health in Networks Study, Baltimore, MD, 2018 (N=245).

Variable			Main partners					Nonmain partners		
			Unadjusted prevalence ratio (95% CI)		Adjusted prevalence ratio (95% CI)^a^		Unadjusted prevalence ratio (95% CI)			Adjusted prevalence ratio (95% CI)^b^
Discussed HIV results with the most recent respective main or nonmain partner (reference “No”)			1.57 (1.29-1.92)^c^		1.46 (1.21-1.75)^c^		1.77 (1.40-2.24)^c^			1.54 (1.23-1.93)^c^
**Age (by quartile group; years)**										
	18-25	1.24 (1.04-1.48)^d^		1.06 (0.90-1.25)		0.99 (0.77-1.28)			0.78 (0.62-0.99)^d^	
	26 to 29	1.12 (0.92-1.36)		1.04 (0.87-1.24)		0.88 (0.67-1.17)			0.76 (0.59-0.99)^d^	
	30 to 34	0.92 (0.72-1.17)		0.86 (0.69-1.05)		0.87 (0.65-1.17)			0.79 (0.61-1.02)	
	35 to 45 (reference)	N/A^d^		N/A		N/A			N/A	
**Race**										
	Black (reference)	N/A		N/A		N/A			N/A	
	White	1.20 (1.05-1.36)^e^		1.06 (0.94-1.20)		1.36 (1.12-1.65)^e^			1.11 (0.92-1.34)	
	Other	1.04 (0.81-1.36)		1.02 (0.79-1.34)		1.07 (0.73-1.59)			0.86 (0.57-1.31)	
Living with partner (reference “No”)			1.28 (1.15-1.43)^c^		1.26 (1.13-1.40)^c^		N/A			N/A
STI^f^ (reference “No”)			N/A		N/A		0.76 (0.55-1.04)			0.95 (0.72-1.26)
HIV (reference “No”)			0.71 (0.59-0.84)^e^		0.78 (0.67-0.91)^d^		0.61 (0.47-0.78)^e^			0.70 (0.55-0.88)^d^
Standardized EDC^g^ Score			1.18 (1.07-1.29)^d^		1.15 (1.06-1.25)^d^		1.33 (1.17-1.50)^e^			1.27 (1.13-1.43)^e^

^a^Model adjusted for age, standardized EDC score, race, living with partner, and living with HIV.

^b^Model adjusted for age, standardized EDC score, race, STI diagnosis at 3-month visit, and living with HIV.

^c^*P*<.001.

^d^*P*<.05.

^e^*P*<.01.

^f^STI: sexually transmitted infection.

^g^EDC: enhancing dyadic communication.

## Discussion

### Principal Findings

Given the high burden of HIV, gonorrhea, and syphilis cases among Black sexual minority men in the United States, there is an urgent need to identify innovative digital health care strategies to reduce disparities. This study aimed to determine desired patient portal features that may be used to address STI/HIV prevention and care among urban sexual minority men and measure the associations between discussing HIV test results and willingness to use patient portals in sharing STI/HIV test results with the most recent sex partners. In this study, several patient portal features to combat the STI/HIV burden were endorsed, including (1) features to help manage HIV care for sexual minority men living with HIV, (2) services to retrieve resources and information on PrEP, PEP, and educational information about STIs, and (3) access to validate STI/HIV test information to facilitate the exchange of test results with sex partners. A history of discussing HIV test results with recent partners was previously unexplored as a factor in willingness to disclose web-based STI/HIV test results. Sexual minority men reporting discussions with sex partners regarding test results may be recruited as early adopters and diffusers of patient portal-facilitated exchanges of STI testing information.

### EDC and Willingness to Use Patient Portals for Sharing Test Results

This study confirms the criterion validity of EDC in measuring the psychosocial construct associated with an individual’s willingness to use patient portals to disclose test results with their sex partners, newly in a sample of predominantly Black sexual minority men. The EDC scale may be used to estimate a patient’s or study participant’s likelihood of leveraging patient portals for STI/HIV test result disclosures with sex partners as a function of what they perceive to be the advantages of the behavior. EDC data may be used to tailor messaging aimed to intervene on or support decisions to facilitate patient portal test result disclosures with partners. Compared to the 2018 American Men’s Internet Survey data (sample mean EDC score 7.55, SD 1.72), the mean EDC scores in this study sample were lower [35]. Similarly, a lower proportion of participants in this study were willing to disclose web-based STI/HIV test results with both main and nonmain partners, compared to 2018 American Men’s Internet Survey participants [28]. These findings coincide with racial differences in willingness to disclose web-based test results. Although nonsignificant, White sexual minority men were more inclined to disclose results using patient portals in this study than other races. Greater HIV prevalence among Black participants than among White participants may have contributed to differences by race or ethnicity in the willingness to disclose web-based test results, since HIV-related stigma commonly results in predilections toward restricting disclosure [36,37]. When looking solely at participants not living with HIV, proportional differences in willingness to disclose test results by race or ethnicity were reduced in this study. Greater willingness to disclose STI/HIV test results with main partners using patient portals among younger than among older participants is consistent with previous studies [27,28]. However, differences by age were diminished when considering willingness to disclose to nonmain partners.

### Empowerment and Social Determinants

Patient portals serve a valuable role in bolstering access to health care information and services. Since Black patients are more likely than White patients to access patient portals using mobile devices compared to using a computer, the design of culturally appropriate patient portal interventions should include patient-facing interfaces that are also mobile device friendly [38]. Empowerment of Black sexual minority men to combat the STI/HIV burden includes building the versatile capacity of patient portals according to their expressed desires for sexual health utility. As patient portal end users, their voices must influence the design of features. Notably, portals could include interventions tailored to help persons at risk for HIV overcome barriers to PrEP uptake, such as intersectional stigma, fear of side effects, poor patient-provider communication, and low HIV risk perception [22,23]. Portals could allow sexual minority men living with HIV to access and share information about their most recent viral load with partners, informing decision-making. Portals can also be used to disseminate educational information for partners of sexual minority men living with HIV, including the definitive finding that people living with HIV who have achieved sustained viral suppression cannot transmit HIV through sex or U=U [14]. Future research could examine ways sexual minority men living with HIV can use patient portals to deliver information about U=U and their undetectable viral load to potential sexual partners and whether portal-facilitated disclosures might reduce anxiety, awkwardness, or fear of disclosure [16].

Previous research showed that game-based elements in a web-based intervention focused on delivering information and resources and social support effectively reduced condomless anal sex among young Black sexual minority men [39]. However, contemporary studies suggest that web-based gamification toward HIV prevention may be most valued outside the patient portal system [11,35]. Tools for managing HIV are of importance to sexual minority men [11]. However, there are concerns to be addressed in the design and implementation of patient portal modules for patients living with HIV, including breach of confidentiality and reduced human interaction with health care providers [40,41]. Key features expressly valued by study participants for improving HIV prevention and care, including partner notification and tips for communicating with sex partners, are missing components in the currently available patient portal systems. Today, there remains tremendous opportunities and benefits yet to be realized by building patient portals that are responsive to the preferences and needs of unique patient populations [42].

The backbone of STI/HIV field prevention services is contact tracing, in which potentially exposed sexual or drug injection partners are anonymously contacted to inform them of their risk for STI/HIV and provide them with testing, counseling, and referrals for other services [43]. Out of the COVID-19 pandemic emerged innovative methods for contact tracing using mobile apps [44,45]. Future research may explore the potential for patient notifications of potential STI/HIV exposures through the patient portal. Including HIV genetic subtype or phylogenetic information in the patient portal record was also indicated as a valued feature by most of the current study’s sample of urban sexual minority men. Providing molecular information in a patient-facing direction may take on different beneficial characteristics to urban sexual minority men compared to HIV molecular surveillance programs which have been criticized as potentially having a role in HIV criminalization cases [45,46].

### Limitations

There are several limitations to this study. Participants comprise a convenience sample from one Mid-Atlantic city, and thus there is vulnerability to sample bias and unknown generalizability to sexual minority men in this setting and in other urban settings. Sampling was, however, intentional to include clinic and non–clinic-based community settings to improve generalizability. Cross-sectional data do not allow for causal inferences. In addition, results may be influenced by recall and social desirability biases [47,48]. ACASI was used to reduce the impact of social desirability bias, particularly for sensitive information.

### Conclusions

Patient portals are an extension of health care institutions and thus may serve as a trusted source for educational information related to sexual health. The expressed desires among the majority Black sexual minority men in this study provide guidance on how patient portals may be leveraged as tools for STI/HIV prevention. Perhaps unsurprisingly, the strong association between anticipated willingness to use patient portals to share STI/HIV information and report of prior discussion of HIV test results with partners suggests that one’s history of discussing HIV results is a major component of willingness to disclose web-based test results. Patient portals may bridge the gap by delivering interventions that provide information that empowers Black sexual minority men across health systems rife with health inequities. Future studies should leverage patient-level data on past HIV test results, discussion behavior with partners, the EDC psychosocial construct, and age in tailoring interventions focused on increasing disclosure events between sex partners and reducing risk factors associated with STI/HIV transmission, particularly among sexual minority men living with HIV.

## Abbreviations

ACASI: audio-computer self-assisted interview
aPR: adjusted prevalence ratio
ART: antiretroviral therapy
EDC: enhancing dyadic communication
PEP: postexposure prophylaxis
PrEP: preexposure prophylaxis
RDS: respondent driven sampling
STI: sexually transmitted infection
USHINE: Understanding Sexual Health in Networks
U=U: undetectable equals untransmittable

## Data Availability

Data sets are currently unavailable to be publicly shared due to limitations of the current data agreement.
