# Correction: Desires for Individual- and Interpersonal-Level Patient Portal Use for HIV Prevention Among Urban Sexual Minority Men: Cross-sectional Study

**DOI:** 10.2196/46774

**Published:** 2023-03-08

**Authors:** Kevon-Mark P Jackman, Carla Tilchin, Jessica Wagner, Ryan E Flinn, Maria Trent, Carl Latkin, Sebastian Ruhs, Errol L Fields, Matthew M Hamill, Carlos Mahaffey, Adena Greenbaum, Jacky M Jennings

**Affiliations:** 1 Division of Adolescent and Young Adult Medicine Department of Pediatrics Johns Hopkins University School of Medicine Baltimore, MD United States; 2 Center for Child and Community Health Research Department of Pediatrics Johns Hopkins School of Medicine Baltimore, MD United States; 3 Department of Health, Behavior, and Society Johns Hopkins Bloomberg School of Public Health Baltimore, MD United States; 4 Medical College of Georgia Augusta University Augusta, GA United States; 5 Chase Brexton Health Services Baltimore, MD United States; 6 Division of Infectious Diseases Johns Hopkins School of Medicine Baltimore, MD United States; 7 STI/HIV Program Baltimore City Health Department Baltimore, MD United States; 8 Department of Public Health College of Health and Human Sciences Purdue University West Lafayette, IN United States; 9 Department of Epidemiology Johns Hopkins Bloomberg School of Public Health Baltimore, MD United States

In “Desires for Individual- and Interpersonal-Level Patient Portal Use for HIV Prevention Among Urban Sexual Minority Men: Cross-sectional Study” (JMIR Form Res 2023;7:e43550) the authors noted one error.

The name of the corresponding author appeared within the citation information as:

P Jackman K

This will be changed to read as:

Jackman KP

The correction will appear in the online version of the paper on the JMIR Publications website on March 8, 2023 together with the publication of this correction notice. Because this was made after submission to PubMed, PubMed Central, and other full-text repositories, the corrected article has also been resubmitted to those repositories.

